# Circulating angiopoietin-1 and angiopoietin-2 in critically ill patients: development and clinical application of two new immunoassays

**DOI:** 10.1186/cc6966

**Published:** 2008-07-29

**Authors:** Alexander Lukasz, Julian Hellpap, Rüdiger Horn, Jan T Kielstein, Sascha David, Hermann Haller, Philipp Kümpers

**Affiliations:** 1 Department of Nephrology, Hannover Medical School, Carl-Neuberg-Straße 1, 30625 Hannover, Germany; 2 Department of Gastroenterology, Hepatology and Endocrinology, Hannover Medical School, Carl-Neuberg-Straße 1, Hannover 30625, Germany

## Abstract

**Introduction:**

In critically ill patients, the massive release of angiopoietin-2 (Ang-2) from endothelial Weibel–Palade bodies interferes with constitutive angiopoietin-1 (Ang-1)/Tie2 signaling in endothelial cells, thus leading to vascular barrier breakdown followed by leukocyte transmigration and capillary leakage. The use of circulating Ang-1 and Ang-2 as novel biomarkers of endothelial integrity has therefore gained much attention. The preclinical characteristics and clinical applicability of angiopoietin immunoassays, however, remain elusive.

**Methods:**

We developed sandwich immunoassays for human Ang-1 (immunoradiometric sandwich assay/immunoluminometric sandwich assay) and Ang-2 (ELISA), assessed preanalytic characteristics, and determined circulating Ang-1 and Ang-2 concentrations in 30 healthy control individuals and in 94 critically ill patients. In addition, Ang-1 and Ang-2 concentrations were measured in 10 patients during a 24-hour time course with respect to interference by intravenous antibiotic treatment and by extended daily dialysis.

**Results:**

The assays had detection limits of 0.12 ng/ml (Ang-1) and 0.2 ng/ml (Ang-2). Inter-assay and intra-assay imprecision was ≤8.8% and 3.7% for Ang-1 and was ≤4.6% and 5.2% for Ang-2, respectively. Angiopoietins were stable for 24 hours and were resistant to four freeze–thaw cycles. Angiopoietin concentrations were not associated with age, body mass index or renal function in healthy individuals. Ang-1 and Ang-2 concentrations correlated with severity of illness in critically ill patients. Angiopoietin concentrations were not influenced by antibiotic treatment or by extended daily dialysis.

**Conclusion:**

Ang-1 and Ang-2 might serve as a novel class of biomarker in critically ill patients. According to preclinical and clinical validation, circulating Ang-1 and Ang-2 can be reliably assessed by novel immunoassays in the intensive care unit setting.

## Introduction

Endothelial activation denotes a devastating key event in sepsis pathophysiology that is characterized by increased expression of luminal adhesion molecules, leukocyte recruitment, and altered vasomotor tone, resulting in vascular barrier breakdown [1-3]. The endothelial-specific angiopoietin–Tie ligand–receptor system has recently emerged as a nonredundant regulator of endothelial activation [4-6]. Angiopoietin-1 (Ang-1) and angiopoietin-2 (Ang-2) are antagonistic ligands that bind to the extracellular domain of the Tie2 receptor, which is almost exclusively expressed by endothelial cells. Binding of Ang-1 to Tie2 promotes vessel integrity, inhibits vascular leakage and suppresses inflammatory gene expression [7,8]. Ang-2 is stored in Weibel–Palade bodies and is rapidly secreted and induced upon stimulation, whereas Ang-1 is constitutively expressed by pericytes and vascular smooth muscle cells [5,9,10]. Binding of antagonistic Ang-2 completely disrupts protective Tie2 signaling in the majority of experimental studies [7,11,12]. Ang-2 has also been identified as a Tie2 agonist, however, especially when administered in a supramaximal dose [13,14].

Several pilot studies suggest that measuring circulating Ang-1 and Ang-2 in critically ill patients might provide valuable information on vascular barrier properties. A marked imbalance of the angiopoietin–Tie system in favor of Ang-2 was detected consistently in critically ill patients [15-19]. Elevated Ang-2 concentrations correlate with severity of illness as assessed by the injury severity score [15], the organ failure index [17], and the Acute Physiology and Chronic Health Evaluation (APACHE) II score or Sequential Organ Failure Assessment (SOFA) score [16,18,19]. Circulating Ang-2 predicted outcome in two studies [15,16]. Circulating Ang-2 and the respective Ang-2/Ang-1 ratio therefore constitute potential new biomarkers for endothelial activation in critical illness.

Preanalytic performance, detailed assay characteristics, and clinical applicability of Ang-1 and Ang-2 immunoassays have not been reported. The aim of the present study was to develop, characterize and validate immunoassays for the detection of circulating Ang-1 and Ang-2.

## Materials and methods

### Angiopoietin-1 immunoradiometric sandwich assay

A polyclonal anti-human Ang-1 affinity-purified goat IgG antibody (PAB) and a monoclonal anti-human Ang-1 mouse antibody were obtained from R&D Systems (Minneapolis, MN, USA). Recombinant human Ang-1 (90% purity recombinant, expressed in a murine nonsecreting NSO myeloma cell line) was purchased from Sigma-Aldrich (Munich, Germany).

Maxisorp Startubes (Nunc, Roskilde, Denmark) were coated for 2 hours at 4°C with 0.5 μg/tube monoclonal anti-human Ang-1 mouse antibody in 0.1 M sodium carbonate buffer (pH 9.5), and were then washed twice with phosphate-buffered saline with 0.05% Tween-20 (PBST). Serum samples (100 μl) were then diluted 1:1 with assay buffer (30 g/l BSA, 10 g/l bovine IgG, 1% goat serum, 0.1% NaN_3_, 1 M NaCl, 40 mM sodium phosphate buffer, pH 7.4), were added to the tubes, and were incubated for about 24 hours at 4°C.

PAB was iodinated with ^125^Iod (Hartmann, Braunschweig, Germany) using IODO-GEN (Perbio Science, Bonn, Germany). Unbound ^125^I was separated by desalting on a 10 ml Sephadex G-25 column (Pharmacia, Uppsala, Sweden). The tubes were washed twice with PBST. Two hundred microliters of assay buffer containing 10 ng ^125^I-iodinated PAB (specific activity approximately 0.74 MBq/μg) (tracer) were added to each tube, and were incubated for 4 hours at room temperature. After three washing steps, bound radioactivity was quantified in a gamma counter (LKB Wallac 1261; Perkin-Elmer, Waltham, Massachusetts, USA).

In each experiment, a standard curve was generated with various dilutions of Ang-1. The curve was then used to calculate the Ang-1 concentrations in individual samples.

### Angiopoietin-1 immunoluminometric sandwich assay

In the case of immunoluminometric detection, PAB was conjugated with Acridinium C2 NHS Ester (Assay Designs, Ann Arbor, MI, USA). The conjugated PAB was then quantified in a System Luminometer (Nichols Institute Diagnostics, San Juan Capistrano, California, USA).

### Angiopoietin-2 ELISA

Ang-2 was measured using antibodies included in the DuoSet methodology ELISA (R&D Systems). Recombinant human Ang-2 (95% purity, murine nonsecreting NSO derived; R&D Systems) served as the standard.

ELISA plates (Nunc Maxisorb, Roskilde, Denmark) were coated overnight at 4°C with 2 μg/ml monoclonal Ang-2 antibody in 0.1 M sodium carbonate buffer (pH 9.5), and were then washed three times with 300 μl PBST. Serum samples (50 μl) were then diluted 1:1 with assay buffer 1 (30 g/l BSA, 10 g/l bovine IgG, 1% goat serum, 0.1% NaN_3_, 1 M NaCl, 40 mM sodium phosphate buffer, pH 7.4), were added to the tubes, and were incubated for 2 hours at room temperature on an orbital shaker. After removal of the serum samples, the tubes were washed three times with PBST. One hundred microliters of assay buffer 2 (0.5% BSA, 1% mouse serum, 0.15 M NaCl, 40 mM sodium phosphate buffer, 0.1% Thimerosal, pH 7.4) containing 1 μg/ml biotinylated anti-Ang-2 antibody were added to each tube, and were incubated for 4 hours at room temperature. After three washing steps, 100 μl streptavidin in assay buffer 2 were added to each tube, and were incubated for 20 minutes at room temperature. After three final washing steps with PBST, 100 μl substrate solution (10 mg tetramethylbenzidine in 10 ml of 0.1 M citrate buffer, pH 5, 4 μl H_2_O_2_) were added to each tube and were incubated for 15 minutes. The assay was stopped by sulfuric acid (1 M H_2_SO_4_) and was measured using a microplate reader (Tecan spectra mini; Tecan, Crailsheim, Germany).

### Healthy control individuals

To assess the detection limits and precision, the interference, and the preanalytic performance of the Ang-1 immunoradiometric sandwich assay (IRMA)/immunoluminometric sandwich assay (ILMA) and of the Ang-2 ELISA, we obtained serum samples from 30 apparently healthy medical students and employees at Hannover Medical School (17 males, 13 females; age, 59 years (27 to 75 years); body mass index, 25 kg/m^2 ^(19 to 32 kg/m^2^); serum creatinine, 81.7 μmol/l (53.9 to 91.1 μmol/l); estimated glomerular filtration rate (MDRD (Modification of Diet in Renal Disease)formula), 81 ml/min (56 to 107 ml/min)). All individuals provided written informed consent, and the institutional review board of Hannover Medical School approved the study (No. 4373).

### Critically ill patients and study protocol

To validate the immunoassays in a clinical setting, Ang-1 and Ang-2 concentrations were measured in sera from 94 Caucasian medical intensive care unit (ICU) patients (Table 1) and were correlated with SOFA scores [20]. Patients with a history of diabetes mellitus were excluded from the present study.

**Table 1:** Demographic and clinical characteristics of patients

Characteristic	Value
Number of patients	94
Male	56
Female	48
Age (years)	59 (21 to 69)
Reason for medical intensive care unit admission	
Abdominal	35 (37%)
Pulmonary	27 (29%)
Urogenital/retroperitoneal	8 (9%)
Bloodstream infections	6 (6%)
Cerebrovascular	8 (9%)
Miscellaneous	10 (11%)
Mean arterial pressure (mmHg)	67 (23 to 96)
Mechanically ventilated	78 (83%)
PaO_2_/FiO_2 _(mmHg)	240 (48 to 646)
Adrenaline or noradrenaline	
None	32 (34%)
≤ 0.1 μg/kg/min	19 (20%)
>0.1 μg/kg/min	43 (46%)
Sequential Organ Failure Assessment score	13 (3 to 22)

Data presented as *n *(%) or median (range).

Patients were recruited at Hannover Medical School, a tertiary care university hospital. Enrollment was performed after obtaining written informed consent from the patient or his/her legal representatives. If the patient was recovering and able to communicate, he/she was informed of the study purpose and consent was required to further maintain status as a study participant. The study was carried out in accordance with the declaration of Helsinki and was approved by the institutional review board (No. 4373).

In 10 critically ill patients, serial measurements (Ang-1 and Ang-2) were performed during a 24-hour period. Inclusion criteria were age ≥18 years, the need for extended daily dialysis (EDD), and the need for antibiotic treatment. The approach was chosen to study circadian variations of Ang-1 and Ang-2 in the ICU setting, to study interference with antibiotic treatment, and to study absorption/clearance via EDD in the same patient while avoiding interpatient variability.

Moxifloxacin (400 mg) and ertapenem (1 g) were infused intravenously during a period of 60 minutes. Blood samples were drawn from the arterial line placed in the radial artery or femoral artery 0, 0.5, 1, 2, 4, 6, 8, 10, 12, 16, 20 and 24 hours after administration of antibiotics. EDD was started 8 hours after administration of antibiotics, using the GENIUS batch dialysis system (Fresenius Medical Care, Bad Homburg, Germany) with a polysulfone high-flux dialyzer (F60S; Fresenius Medical Care) as described previously [21-23]. EDD was performed over an 8-hour period, and the blood and countercurrent dialysate flow rate was maintained at 160 ml/min in all subjects. Vascular access in all patients was achieved by a double-lumen catheter inserted either into the internal jugular or into the femoral vein. Extra blood samples were drawn before and after dialysis (that is, from the afferent artery and efferent venous dialyzer blood tubing) to calculate the dialyzer clearance from the predialyzer and postdialyzer concentration difference and the estimated plasma flow. Blood water clearance (*CL*_ang_) of angiopoietins across the dialyzer was calculated from arterial (*C*_a_) and venous (*C*_v_) angiopoietin concentrations, the ultrafiltration rate (*Q*_f_), and the blood water flow rate (*Q*_a_) using the following equation: *CL*_ang _= [*C*_a _× *Q*_a _- *C*_v _× (*Q*_a _- *Q*_f_)]/*C*_a_.

### Statistical analysis

Differences between patients and healthy control individuals and between venous and arterial angiopoietin concentrations were evaluated using the nonparametric two-sided Mann–Whitney rank sum test. Friedman's test followed by Dunn's correction for multiple testing was used to detect statistical differences in angiopoietin concentrations during 24-hour follow-up. Differences between angiopoietin concentrations in patients with cardiovascular disease or malignancies were compared with matched critically ill control individuals by the paired Wilcoxon signed-rank test. Correlations between variables were assessed by the Spearman rank correlation coefficient (Ang-2). Pearson's correlation coefficient and linear regression analysis was performed after logarithmic transformation of angiopoietin concentrations (log_Ang_).

Statistical significance was accepted at 5% probability concentrations. Data are displayed as the median and range (minimum to maximum) unless otherwise stated. All statistical analyses were performed with the SPSS package (SPSS Inc., Chicago, IL, USA) and with GraphPad Prism software (GraphPad Prism Software Inc. San Diego, CA, USA).

## Results

### Detection limits and precision

The detection limit of the Ang-1 IRMA, calculated as the mean ± three standard deviations for 10 replicate measurements of the zero standard (calibrator free of analyte), was 0.12 ng/ml. The within-run (intra-assay) coefficient of variation, determined by measuring three serum samples in 15 parallel measurements, ranged from 1.9% to 3.7% for samples containing 61.5 ng/ml (58.7 to 67.5 ng/ml) Ang-1. The total (inter-assay) coefficient of variation was determined by measuring two serum samples in eight assay runs on different days, by two different operators, and with different lots of tubes, tracer, and calibrator. The inter-assay imprecision was 8.4% and 8.8% for samples containing 1.7 ng/ml (1.5 to 1.9 ng/ml) Ang-1 and 21.8 ng/ml (17.9 to 22.9 ng/ml) Ang-1.

**Figure 1:**
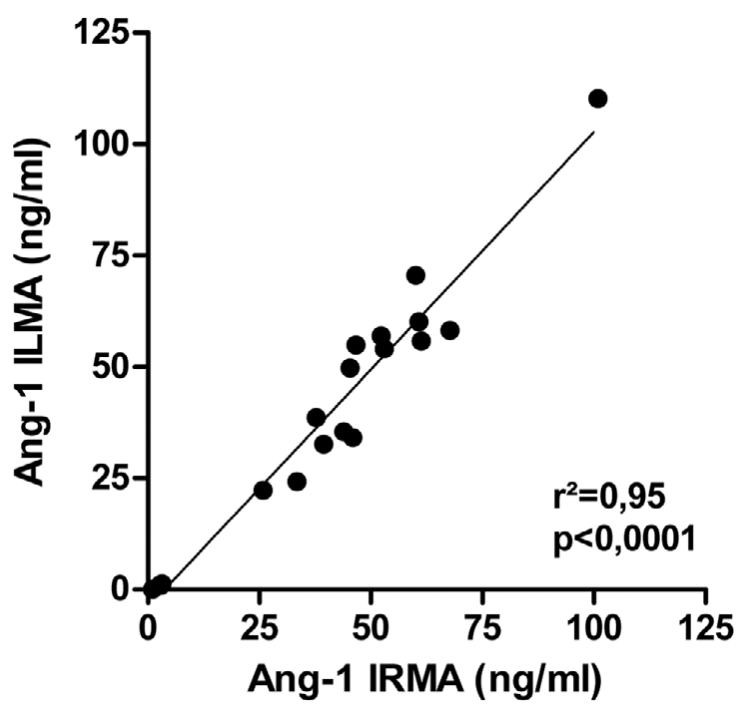
Correlation of angiopoietin-1 concentrations measured by immunoluminometric and immunoradiometric methods. Twenty serum samples were analyzed for the angiopoietin-1 (Ang-1) concentration by immunoluminometric sandwich assay (ILMA) and by immunoradiometric sandwich assay (IRMA). Correlation between both methods was excellent (*P *< 0.0001, *r*^2 ^= 0.95).

We also evaluated the immunoluminometric (ILMA) Ang-1 detection instead of using the immunoradiometric method to simplify and accelerate test performance. Twenty serum samples were analyzed by ILMA and by IRMA respectively. Correlation between both methods was excellent (*P *< 0.0001, *r*^2 ^= 0.95) (Figure 1).

The detection limit of the Ang-2 ELISA was 0.2 ng/ml. The intra-assay coefficient of variation for Ang-2, determined by measuring three serum samples in eight parallel measurements, ranged from 2.0% to 5.2% for samples containing 2.0 ng/ml (0.7 to 4.1 ng/ml) Ang-2. The inter-assay imprecision, determined in analogy to the Ang-1 IRMA, was 3.9% and 4.6% for samples containing 3.6 ng/ml (3.4 to 3.8 ng/ml) Ang-2 and 7.2 ng/ml (6.9 to 7.7 ng/ml) Ang-2.

### Specificity

To test for potential cross-reactivity of Ang-1 with Ang-2, we added 100 ng/ml recombinant human Ang-1 (or recombinant human Ang-2 respectively) to three serum samples obtained from two apparently healthy individuals and from one critically ill patient. No cross-reactivity between Ang-1 and Ang-2 was observed (*P *= 0.9 and *P *= 0.87, respectively).

### Interference studies

To assess whether unrelated biological substances interfere with the Ang-1 and Ang-2 immunoassays, we added potentially interfering substances to four serum samples. Paired Wilcoxon testing indicated that the assay was not appreciably influenced by albumin (up to 40 g/l) or by heparin (up to 400,000 U/l). The Ang-1 and Ang-2 values obtained for samples with and without added interfering substances differed by <20% in all cases.

### Preanalytic performance

#### Difference between serum and plasma samples

**Figure 2:**
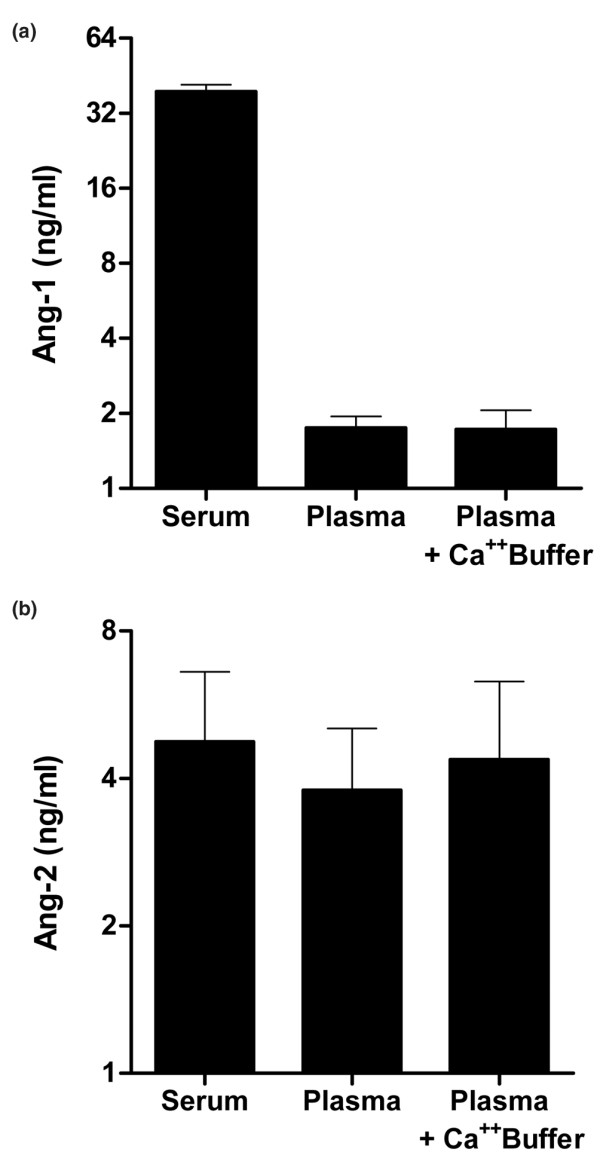
Comparison between detection of angiopoietin-1 and angiopoietin-2 concentrations in serum and plasma. **(a) **Angiopoietin-1 (Ang-1) and **(b) **angiopoietin-2 (Ang-2) concentrations were determined in parallel in serum and in ethylenediamine tetraacetic acid (EDTA) plasma samples obtained from the same five individuals. Importantly, the choice of anticoagulant matrix has a marked influence on Ang-1 measurement. Ang-1 was hardly detectable in EDTA-treated plasma, irrespective of the choice of buffer (calcium-supplemented buffer versus normal assay buffer; see Materials and methods). Ang-2 concentrations obtained from EDTA-treated plasma were lower (~80%) compared with values obtained from serum (100%). In contrast to Ang-1, the use of a calcium-supplemented buffer instead of the normal assay buffer could abolish the difference between serum and plasma for Ang-2.

We analyzed angiopoietin concentrations in parallel in serum samples and in ethylenediamine tetraacetic acid (EDTA) plasma samples obtained from the same five individuals. Of note, Ang-1 was hardly detectable in EDTA-treated plasma (Figure 2a). This apparent difference between serum and plasma could not be mitigated using a modified, calcium-supplemented buffer (30 g/l BSA, 10 g/l bovine IgG, 1% goat serum, 0.1% NaN_3_, 40 mmol/l CaCl, 20 mmol/l Tris(hydroxymethyl)-aminomethane buffer, pH 7.4) instead of the normal assay buffer.

After correction for sample dilution (EDTA), Ang-2 concentrations obtained from EDTA-treated plasma were lower (~80%) compared with values obtained from serum (100%). In contrast to Ang-1, the use of a calcium-supplemented buffer instead of the normal assay buffer could abolish the difference between serum and plasma (Figure 2b).

#### Preprocessing storage and stability

To test the preprocessing stability, serum samples from seven healthy individuals were stored for up to 24 hours at either room temperature or at 4°C. Storage at both temperatures did not produce a discernible loss at 24 hours of Ang-1 immunoreactivity (107% (96% to 102%) versus 100% at baseline; and 91% (100% to 102%) versus 100% at 24 hours) or of Ang-2 immunoreactivity (92% (90% to 91%) versus 100% at baseline; and 101% (110% to 119%) versus 100% at 24 hours), respectively.

#### Freeze and thaw

Moreover, four cycles of freezing (20 hours at -70°C) and thawing (4 hours at room temperature) induced no discernible loss of Ang-1 immunoreactivity (102% (97% to 107%) versus 100% at baseline) or of Ang-2 immunoreactivity (92% (85% to 105%) versus 100% at baseline) in tests of five serum samples.

#### Dilution series

**Figure 3:**
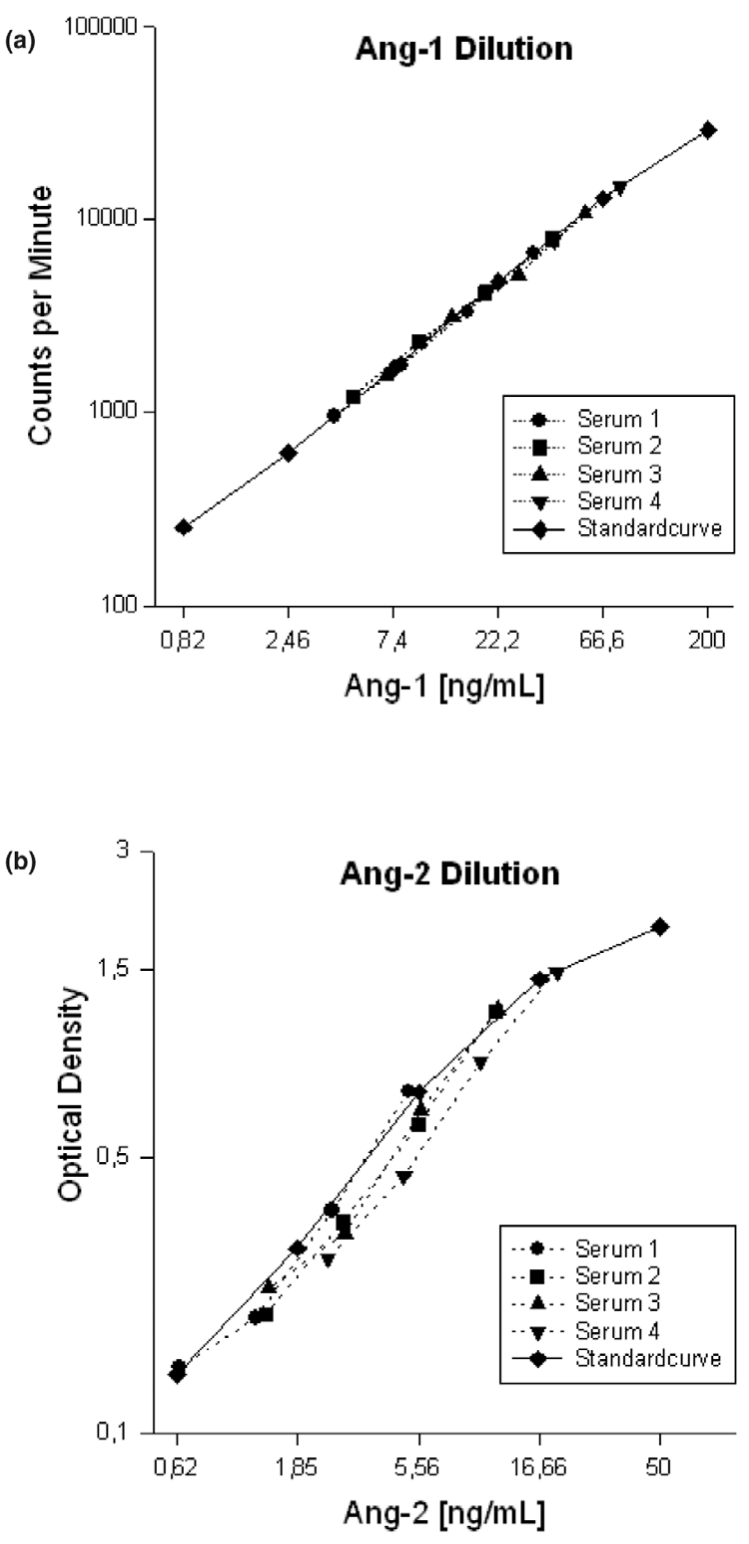
Assay linearity and standard reference curves for angiopoietin-1 and angiopoietin-2 immunoassays. Dilution studies demonstrated both excellent assay linearity as well as adequate parallelism between standard references (recombinant human angiopoietin-1 or recombinant human angiopoietin-2) and serially diluted serum sample curves for **(a) **angiopoietin-1 (Ang-1) and **(b) **angiopoietin-2 (Ang-2) assays.

To test for assay linearity, standard reference curves (recombinant human Ang-1 or recombinant human Ang-2) and serially diluted serum samples from five patients were compared. Dilution studies demonstrated both excellent assay linearity as well as adequate parallelism between standard references and serially diluted serum sample curves for Ang-1 and Ang-2 assays, respectively (Figure 3).

### Association of circulating Ang-1 and Ang-2 concentrations with clinical and laboratory characteristics in healthy control individuals

In healthy control individuals, circulating Ang-1 did not correlate with age (*r *= 0.12, *P *= 0.61), with body mass index (*r *= 0.06, *P *= 0.81), with renal function when tested for serum creatinine (*r *= 0.1, *P *= 0.65) or with estimated glomerular filtration rate (MDRD formula) (*r *= 0.03, *P *= 0.89). Interestingly, Ang-1 concentrations were slightly higher in women (57.6 ng/ml (39.2 to 61.7) ng/ml) compared with men (49.8 ng/ml (43.5 to 39.4) ng/ml) (*P *= 0.025).

Ang-2 concentrations were not associated with age (*r *= 0.04, *P *= 0.86), with body mass index (*r *= 0.06, *P *= 0.81), with renal function (*r *= 0.1, *P *= 0.61 and *r *= 0.09, *P *= 0.71), or with gender (*P *= 0.152).

### Circulating Ang-1 and Ang-2 concentrations correlate with severity of illness in critically ill patients

**Figure 4:**
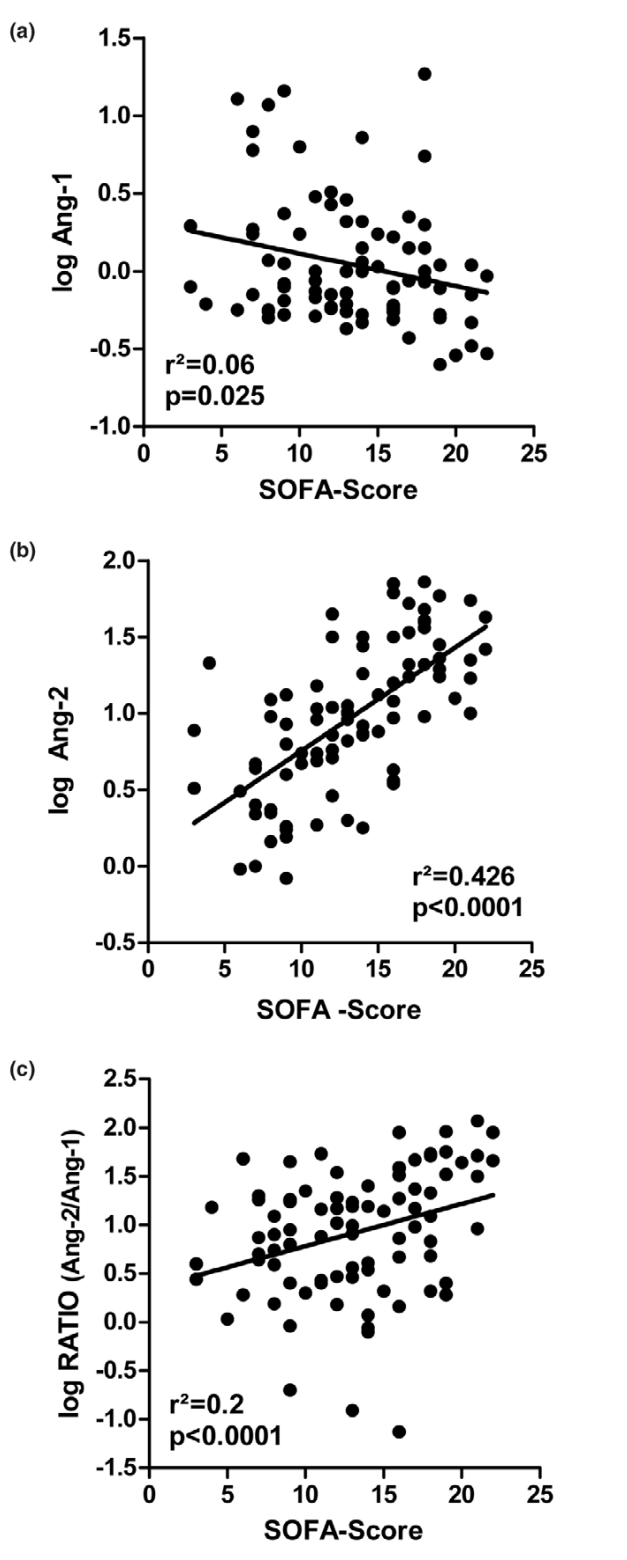
Circulating angiopoietin-1 and angiopoietin-2 concentrations correlate with severity of illness in critically ill patients. Scatter plots showing in 94 critically ill patients the correlation between Sequential Organ Failure Assessment (SOFA) scores and **(a) **circulating angiopoietin-1 (Ang-1) concentrations, **(b) **angiopoietin-2 (Ang-2) concentrations, and **(c) **the Ang-2/Ang-1 ratio.

In 94 critically ill patients, a significant inverse correlation between Ang-1 concentrations and the SOFA score was observed using linear regression (*r*^2 ^= 0.06, *P *= 0.025). A positive correlation was present between the SOFA score and both Ang-2 and the Ang-2/Ang-1 ratio (*r*^2 ^= 0.426, *P *< 0.0001 and *r*^2 ^= 0.2, *P *< 0.0001) (Figure 4). Ang-1 and Ang-2 did not correlate with sex (*P *= 0.4 and *P *= 0.5) or with age (*P *= 0.16 and *P *= 0.7) in critically ill patients.

A subgroup analysis in SOFA score and gender-matched critically ill control patients was performed to compare Ang-1 and Ang-2 concentrations in patients with/without atherosclerotic cardiovascular disease (*n* = 11), and with malignant disease (*n* = 6) respectively. The Ang-1 and Ang-2 concentrations were not different among respective subgroups, as revealed by the paired Wilcoxon signed-rank test (cardiovascular disease, *P *= 0.48 and *P *= 0.19; malignant disease, *P *= 0.7 and *P *= 0.81).

### Circulating Ang-1 and Ang-2 concentrations in 10 critically ill patients during a 24-hour time course

The median Ang-1 concentrations in those 10 critically ill patients (12.0 ng/ml (0.6 to 76.7) ng/ml) were significantly lower compared with healthy control individuals (56.4 ng/ml (34.5 to 71.3 ng/ml) (*P *< 0.0001). In contrast, serum Ang-2 concentrations were markedly elevated in patients (17.7 ng/ml (1.9 to 41.7 ng/ml)) compared with healthy control individuals (0.9 ng/ml (0.3 to 2.6 ng/ml)) (*P *< 0.0001).

**Figure 5:**
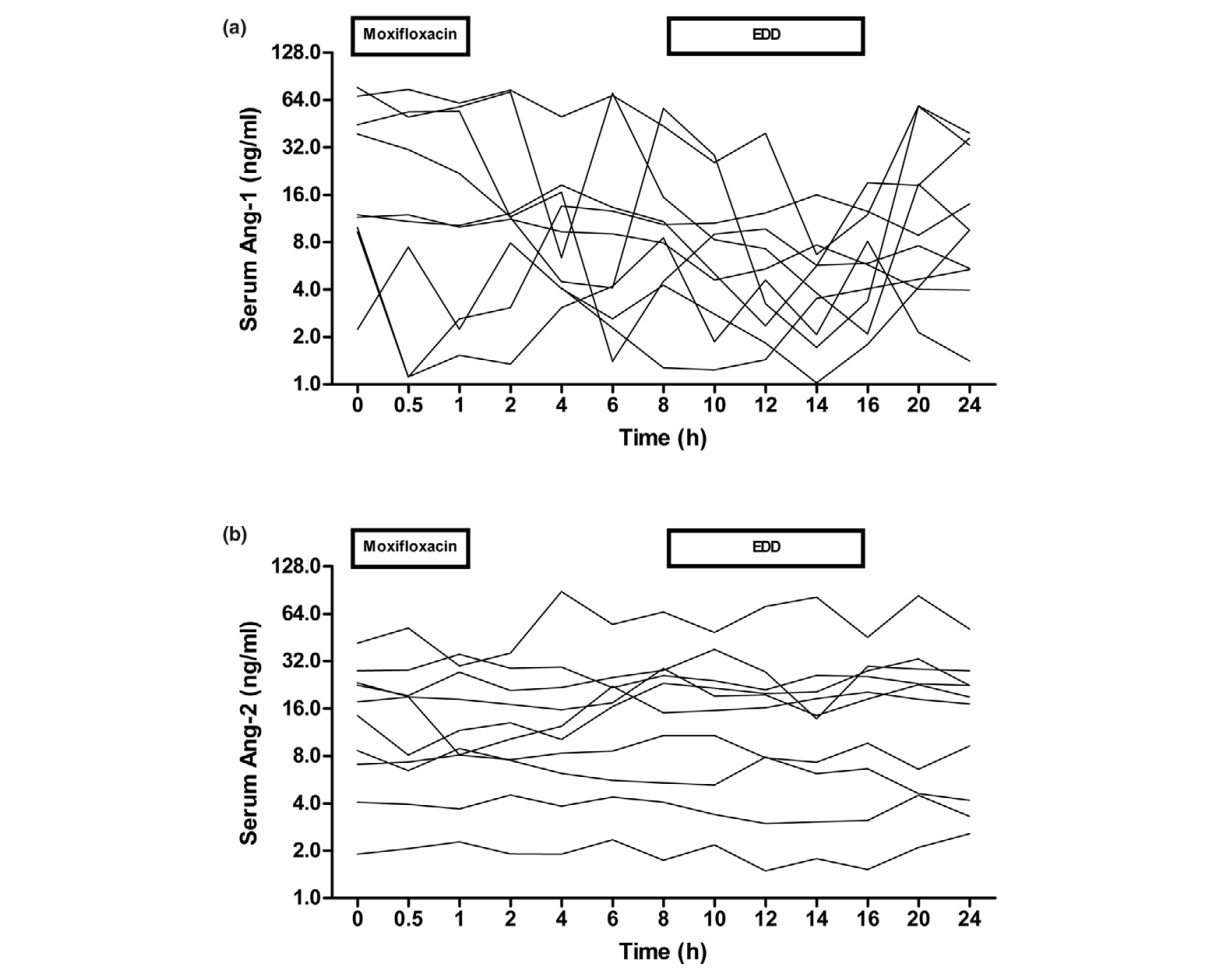
Angiopoietin-1 and angiopoietin-2 concentrations are stable during a 24-hour time course in critically ill patients. Intraindividual **(a) **angiopoietin-1 (Ang-1) serum concentrations showed higher variability within the 24-hour study period than **(b) **angiopoietin-2 (Ang-2) concentrations (*P *= 0.888 and *P *= 0.205). Serum concentrations of Ang-1 and Ang-2 were not altered significantly within 8 hours after the first administration of intravenous moxifloxacin (*P *= 0.885 and *P *= 0.372), or during 8 hours of extended daily dialysis (EDD) (*P *= 0.542 and *P *= 0.423). Each line represents a single patient.

Intraindividual Ang-1 serum concentrations showed higher variability within the 24-hour study period than Ang-2 concentrations (*P *= 0.888 and *P *= 0.205). A higher intraindividual variability of Ang-2 serum concentrations was frequently observed in patients with high Ang-2 concentrations compared with patients with low Ang-2 concentrations (Figure 5).

### Antibiotic treatment

The onset of potent antibiotics can affect cytokine and growth factor concentrations via direct and indirect mechanisms (for example, endotoxemia). Serum concentrations of Ang-1 and Ang-2 were not altered significantly within 8 hours after the first administration of intravenous moxifloxacin/ertapenem cotherapy (*P *= 0.885 and *P *= 0.372) (Figure 5).

### Extended daily dialysis

**Figure 6:**
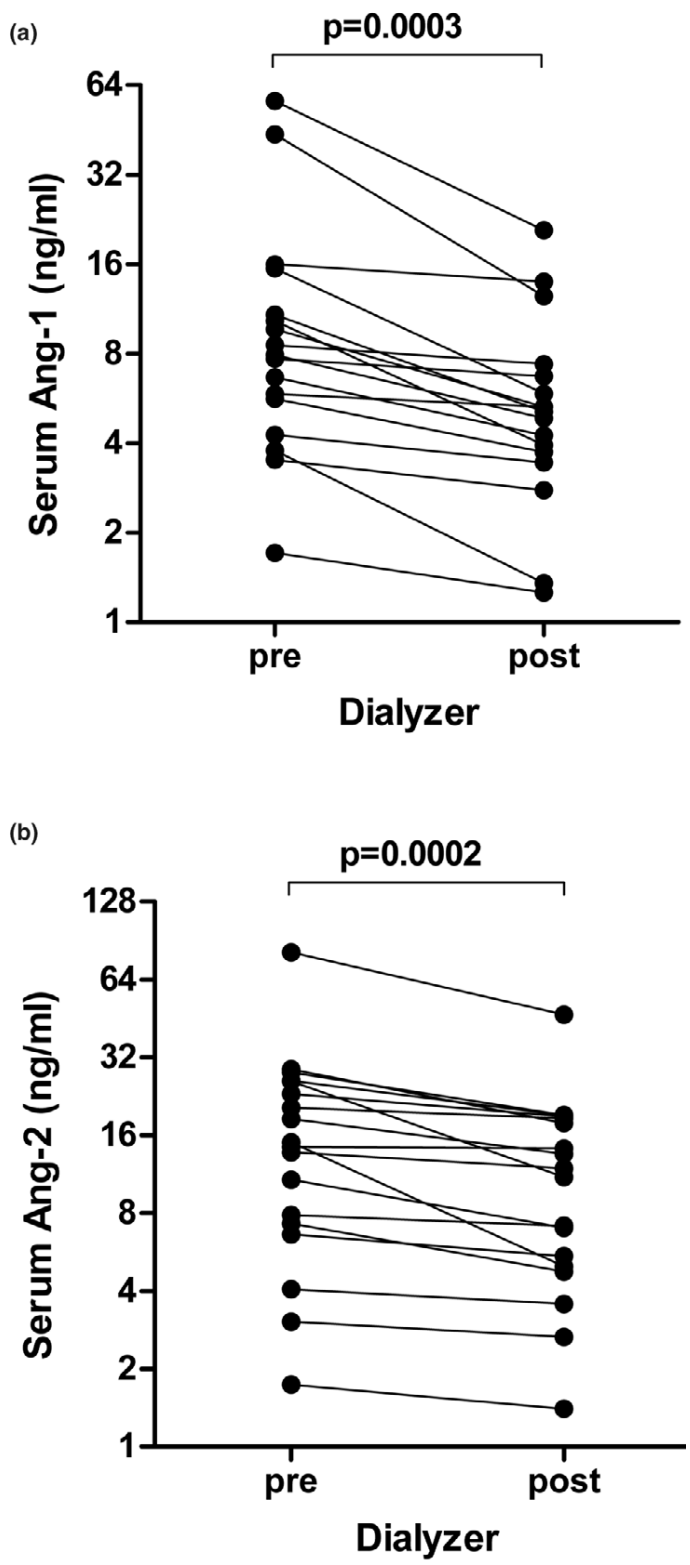
Predialyzer and postdialyzer angiopoietin-1 and angiopoietin-2 concentrations. Aligned dots indicate individual predialyzer and postdialyzer angiopoietin-1 (Ang-1) and angiopoietin-2 (Ang-2) concentrations. **(a) **Median Ang-1 concentrations decreased an average 36.2% from 7.9 ng/ml (1.7 to 56.6 ng/ml) (predialyzer sample) to 5.1 ng/ml (1.3 to 20.8 ng/ml) (postdialyzer sample) (*P *= 0.0003). **(b) **Median Ang-2 concentration decreased by 23.1% from 14.7 ng/ml (1.7 to 81.5 ng/ml) (predialyzer sample) to 11.5 ng/ml (1.4 to 46.9 ng/ml) (postdialyzer sample) (*P *= 0.0002).

It has been shown that several cytokines and angiogenic factors are effectively removed by EDD. Neither Ang-1 nor Ang-2 serum concentrations were significantly lowered during the 8-hour EDD in our patients (*P *= 0.542 and *P *= 0.423) (Figure 5), although Ang-1 and Ang-2 were partially removed by EDD according to the respective predialyzer and postdialyzer angiopoietin concentrations. Median Ang-1 concentrations decreased an average of 36.2% from 7.9 ng/ml (1.7 to 56.6 ng/ml) (predialyzer sample) to 5.1 ng/ml (1.3 to 20.8 ng/ml) (postdialyzer sample) (*P *= 0.0003) (Figure 6a). Likewise, the median Ang-2 concentration decreased by 23.1% from 14.7 ng/ml (1.7 to 81.5 ng/ml) (predialyzer sample) to 11.5 ng/ml (1.4 to 46.9 ng/ml) (postdialyzer sample) (*P *= 0.0002) (Figure 6b).

The calculated clearance during 8 hours of EDD was 57.9 ng/l (15.2 to 114.3 ng/l) dialysate for Ang-1 and was 36.9 ng/l (2.0 to 107.0 ng/l) dialysate for Ang-2. Given a dialysate volume of 75 l, EDD would have removed 15.8% of total circulating Ang-1 and 4.5% total circulating Ang-2 in the absence of a steady state.

## Discussion

The search for biomarkers in critically ill patients that correlate with severity and outcome has a long history. Although biomarkers such C-reactive protein, IL-6, and procalcitonin have been linked to disease severity and outcome, specific biomarkers that indicate endothelial activation/integrity are rare [24-28]. Assessment of circulating angiopoietins, especially Ang-2, has gained much attention since Ang-2 and the Ang-2/Ang-1 ratio presumably reflect the extent of endothelial activation in sepsis and related syndromes [12,15-19].

Only one assay system for human Ang-1 and for Ang-2 has so far been described in the literature. These sandwich ELISAs were invariably used for all of the above-mentioned pilot studies [15-19]. Unfortunately, the preanalytic performances of these angiopoietin assays have not so far been reported. For this reason we have developed, characterized, and validated two new immunosandwich assays for the measurement of circulating human Ang-1 (IRMA/ILMA) and Ang-2 (ELISA). The decisive results are as follows.

First, both assay systems achieved good detection limits of 0.12 ng/ml (Ang-1) and 0.2 ng/ml (Ang-2), allowing for reliable analyte quantification over a wide concentration range.

The second decisive result is that both assays show adequate intra-assay and inter-assay imprecision across the range of values measured in apparently healthy individuals and in critically ill patients in the ICU. Imprecision of our Ang-1 IRMA was comparable with that of the only other ELISA system for the detection of Ang-1 (R&D Systems). Of note, the intra-assay and inter-assay imprecision of our Ang-2 ELISA was even better (~4% versus ~9%) compared with the commercially available ELISA system for the detection of Ang-2.

Third, the assays are not appreciably influenced by unrelated biological substances, such as albumin or heparin, and show no cross-reactivity between Ang-1 and Ang-2.

Fourth, Ang-1 and Ang-2 are stable in serum at room temperature for at least 24 hours and are resistant to at least four freeze–thaw cycles.

Importantly, another result is that the choice of anticoagulant matrix has a marked influence on Ang-1 measurement. Compared with the commercially available Ang-1 ELISA, therefore, our Ang-1 IRMA failed to detect Ang-1 in EDTA-treated or citrated plasma samples.

Finally, dilution studies proved excellent assay linearity as well as adequate parallelism between standard references and serially diluted serum sample curves for Ang-1 and for Ang-2 assays.

To our knowledge, confounding variables that might influence angiopoietin concentrations have not been evaluated previously. In our cohort of 30 apparently healthy individuals, Ang-1 and Ang-2 concentrations were not associated with age, with body mass index, or with renal function. Interestingly, Ang-1 concentrations were slightly higher in women. This gender difference did not persist, however, in critically ill patients.

In a clinical setting, angiopoietin concentrations correlated well with SOFA scores from 94 medical ICU patients (Ang-2 > Ang-2/Ang-1 ratio > Ang-1). This marked imbalance of the Ang/Tie machinery in favor of excessive Ang-2 underscores the concept of Ang-2 being the dynamic component. In contrast, decreased Ang-1 concentrations might reflect impaired maintenance signaling by pericytes. The present study is the first to report a (negative) correlation between severity of illness and Ang-1.

We have to mention that the present study aimed at preanalytic and clinical validation of two novel angiopoietin immunoassays. We did not focus on the diagnostic or prognostic value of circulating Ang-1 and Ang-2 in this cohort of critically ill patients. In an ongoing prospective trial we shall try to delineate the prognostic value of Ang-1 and Ang-2 in combination with established inflammatory markers.

Animal models of endotoxemia have shown that bacterial lipopolysaccharide triggers functional inhibition of the Ang-1/Tie-2 receptor pathway by reducing Ang-1 and Tie-2 expression and inducing Ang-2 concentrations [29,30]. Experimental and clinical studies demonstrated that biologically active bacteria-derived cell wall components might occur after antibiotic treatment [31]. In the present study, no antibiotic-mediated alterations in Ang-1 and Ang-2 serum concentrations were detectable.

EDD not only corrects acid–base/electrolyte homeostasis and extracellular fluid volume, but has also been shown to exert various direct and indirect immunomodulatory effects [32]. Adsorption of cytokines to the synthetic high-flux dialyzer membrane has been recognized as a major mechanism of pyrogen removal in septic patients [33]. Indeed, measurement of predialyzer and postdialyzer angiopoietin concentrations showed a decrease in Ang-1 (36.2%) and Ang-2 (23.1%) concentrations. A molecular weight >55 kDa makes angiopoietins unlikely participants for dialytic clearance [34]. EDD-associated angiopoietin removal therefore most probably occurs due to nonlinear adsorption to the dialyzer membrane. In spite of partial removal from the plasma, the Ang-1 and Ang-2 concentrations remained unchanged. We conclude that angiopoietin concentrations can be safely measured in patients undergoing renal replacement therapy.

Beside their deleterious effect in sepsis and related syndromes, elevated concentrations of circulating angiopoietins may be present in conditions of abnormal angiogenesis (for example, cardiovascular disease, neoplasia) [35-37]. In the present cohort, angiopoietin concentrations were not different in critically ill patients with cardiovascular disease or with malignant diseases compared with matched control individuals. A pre-existent Ang-1 or Ang-2 elevation, however, might be a confounder in patients with lower SOFA scores. Angiopoietin concentrations in the present study were comparable with those of previously published studies [12,15-19].

## Conclusion

Ang-1 and Ang-2 might serve as a novel class of biomarkers in critically ill patients. According to preclinical and clinical validation, circulating Ang-1 and Ang-2 can be reliably assessed by novel immunoassays in the ICU setting.

## Key messages

• Ang-1 and Ang-2 immunoassays achieved good detection limits, and good intra-assay and inter-assay imprecision, allowing for reliable and specific analyte quantification over a wide concentration range.

• Ang-1 and Ang-2 are stable in serum at room temperature for at least 24 hours, are resistant to at least four freeze–thaw cycles, and are not influenced by unrelated biological substances allowing for applicability in the clinical routine.

• In 94 ICU patients, the Ang-1 and Ang-2 concentrations correlated well with SOFA scores and could be reliably assessed during antibiotic treatment and EDD in the ICU.

• Ang-1 and Ang-2 immunoassays could serve as novel readily available tools to assess endothelial activation and impairment in critically ill patients.

## Abbreviations

Ang-1 = angiopoietin-1; Ang-2 = angiopoietin-2; BSA = bovine serum albumin; EDD = extended daily dialysis; EDTA = ethylenediamine tetraacetic acid; ELISA = enzyme-linked immunosorbent assay; ICU = intensive care unit; IL = interleukin; ILMA = immunoluminometric sandwich assay; IRMA = immunoradiometric sandwich assay; PAB = polyclonal anti-human Ang-1 affinity-purified goat IgG antibody; PBST = phosphate-buffered saline with 0.05% Tween-20; SOFA = Sequential Organ Failure Assessment.

## Competing interests

The authors declare that they have no competing interests.

## Authors' contributions

AL and JH contributed equally to the work and are therefore both considered first authors. AL and JH established the immunoassays, performed the experiments, generated the figures and contributed to the manuscript. RH established the immunoassays, supervised the experiments, and analyzed the results. JTK and SD identified patients, provided clinical data, collected samples, participated in the design of the study and reviewed the manuscript. HH supervised the project and reviewed the manuscript. PK had the initial idea, designed and supervised the research, analyzed the results, generated the figures, and wrote the manuscript.
